# Increased risk of pulmonary and extrapulmonary tuberculosis infection in patients with polycystic kidney disease: a nationwide population-based study with propensity score-matching analysis

**DOI:** 10.1186/s12967-021-02921-3

**Published:** 2021-06-09

**Authors:** Ting-Fang Chiu, Tung-Min Yu, Chih-Wei Chiu, Brian K. Lee, Tsuo-Hung Lan, Chi-Yuan Li, Mei-Chen Lin, Chia-Hung Kao

**Affiliations:** 1 Department of Pediatrics, Taipei City Hospital Zhongxiao Branch, Taipei, Taiwan; 2 University of Taipei, Taipei, Taiwan; 3 Department of Education and Research, Taipei City Hospital, Taipei, Taiwan; 4 Graduate Institute of Biomedical Sciences and School of Medicine, College of Medicine, China Medical University, No. 2, Yuh-Der Road, Taichung, 404 Taiwan; 5 Division of Nephrology, Taichung Veterans General Hospital, Taichung, Taiwan; 6 Tsaotun Psychiatric Center, Ministry of Health and Welfare, Nantou, Taiwan; 7 Department of Psychiatry, School of Medicine, National Yang-Ming University, Taipei, Taiwan; 8 Center for Neuropsychiatric Research, National Health Research Institutes, Zhunan, Taiwan; 9 University of Texas, Austin, Dell Seton Medical Center, Austin, USA; 10 Department of Anesthesiology, China Medical University Hospital, Taichung, Taiwan; 11 Department of Post Baccalaureate Medicine, Kaohsiung Medical University, Kaohsiung, Taiwan; 12 Department of Nuclear Medicine and PET Center, China Medical University Hospital, Taichung, Taiwan; 13 Department of Bioinformatics and Medical Engineering, Asia University, Taichung, Taiwan; 14 Center of Augmented Intelligence in Healthcare, China Medical University Hospital, Taichung, Taiwan

**Keywords:** Polycystic kidney disease, Mycobacterium tuberculosis, Lymphopenia

## Abstract

**Background:**

Polycystic kidney disease (PKD) is a common renal disorder affecting approximately 1 in 1000 live births. Tuberculosis (TB) is an infectious disease worldwide. This study investigated the risk of TB infection in patients with PKD.

**Methods:**

A nationwide population-based cohort study was performed using Taiwan’s National Health Insurance Research Database. We used patients’ hospitalization files for the entire analysis during 2000–2012. As per diagnosis, we divided patients into PKD and non-PKD cohorts and the major outcome was TB infection.

**Results:**

A total of 13,540 participants with 6770 patients in each cohort were enrolled. The PKD cohort had a higher risk of TB infection than did the non-PKD cohort after adjusting for age, sex, and comorbidities (adjusted hazard ratio (aHR) = 1.91, 95% confidence interval [CI] = 1.51–2.43). When classifying by sites of pulmonary TB (PTB) and extrapulmonary TB (EPTB), the PKD cohort demonstrated a significantly higher risk of EPTB (aHR = 2.44, 95% CI = 1.46–4.08) as well as a risk of PTB (aHR = 1.69, 95% CI = 1.29–2.22). When stratified by the presence or absence of a comorbidity, high TB infection risk was noted in the PKD patients without any comorbidity (HR = 2.69, 95% CI = 1.69–4.30).

**Conclusions:**

Taken together, our findings suggest that PKD is associated with a 1.91-fold increased risk of TB infection. Medical professionls should maintain a high index of suspicion in daily practice for patients with PKD, particularly those with EPTB infection.

## Background

Polycystic kidney disease (PKD), the most common form of renal disorders, is characterized by gradually enlarged kidneys filled with growing cysts. Its incidence was estimated at 1 in every 400–1000 live births by a study published in 1957 [1]. Recent studies from the United States and Europe have reported that the prevalence of PKD ranges from 3.3 to 4.6 per 10,000 [2–5] and that it is the fourth leading cause of end-stage renal disease (ESRD) after diabetes mellitus, hypertension, and glomerulonephritis in the United States [2].

PKD is the most common congenital renal disease caused by a mutation in the *PKD1* gene on chromosome 16 and *PKD2* gene on chromosome 4 accounting for 85% and 15% of the disease, respectively [6]. The monogenetic mutation leads to significant insufficiency of the phenotype protein polycystin (PC)1 or PC2. Patients with *PKD1* mutations exhibited a more severe phenotype than did those with *PKD2* mutations, and those with *PKD1* mutations present with earlier onset of clinical problems and ESRD [7]. The renal manifestations of PKD include urine-concentrating defect, hematuria, nephrolithiasis, urinary tract infection, flank or abdominal pain, hypertension, proteinuria, chronic kidney disease, and ESRD [8–10]. Approximately 5% of the total number of ESRD cases are caused by PKD in the United States [11, 12].

Patients with PKD were suggested to exhibit various extrarenal manifestations resulting from the gene mutations of PC1 or PC2. The predominant abnormalities are cerebral aneurysms, hepatic and pancreatic cysts, cardiac valve disease, colonic diverticula, abdominal wall and inguinal hernia, seminal vesicle cysts, bronchiectasis, and an increased risk of cancer including renal cell carcinoma [8, 13–16].

Increasing evidence suggests that PKD is associated with underlying immune dysregulation. Mutations of PC1 and PC2 are observed to be associated with decreased proliferation of immortalized lymphoblastoid cells in PKD. PKD is clinically characterized by severe lymphopenia, especially with low blood CD8^+^ T and B lymphocytes, irrespective of kidney function [17].

Tuberculosis (TB) is an emerging infectious disease and is currently prevalent in more than one-third of the world’s population [18]. Host resistance to infection from *Mycobacterium tuberculosis* is significantly mediated by cellular immunity. Among various cells with adaptive immunity, T cells have been considered to play a crucial role in preventing TB infection, and animal studies have proved that CD4 + T cells were involved in providing resistance against *M. tuberculosis* [19]. For example, the increased risk of TB in HIV infection with depleted CD4 + T cells also suggests the importance of these cells in protection against TB.

A study reported that patients with PKD displayed lymphopenia including low counts of T and B cells [17]. In this study, we hypothesized that patients with PKD are more prone to developing TB infection. However, the risk of TB infection in patients with PKD remains unknown. Therefore, this study examined the risk of TB infection in patients with PKD.

## Method

### Data source

In 1995, Taiwan started a single-payer National Health Insurance (NHI) program, and nearly 99.9% of Taiwan’s citizens have been enrolled in this program. The National Health Insurance Research Database (NHIRD) includes the inpatient, outpatient, medical treatment, medication, and other medical service records of patients enrolled in the NHI program. To protect patients’ privacy, their identification numbers are encrypted before releasing the database. In this study, we used hospitalization files for the entire analysis. All patients had at least once hospitalization record with diagnoses defined according to the International Classification of Diseases, Ninth Revision, Clinical Modification (ICD-9-CM) codes. The Research Ethics Committee of China Medical University and Hospital in Taiwan approved the study (CMUH104-REC2-115-AR4).

### Study population

**Fig. 1:**
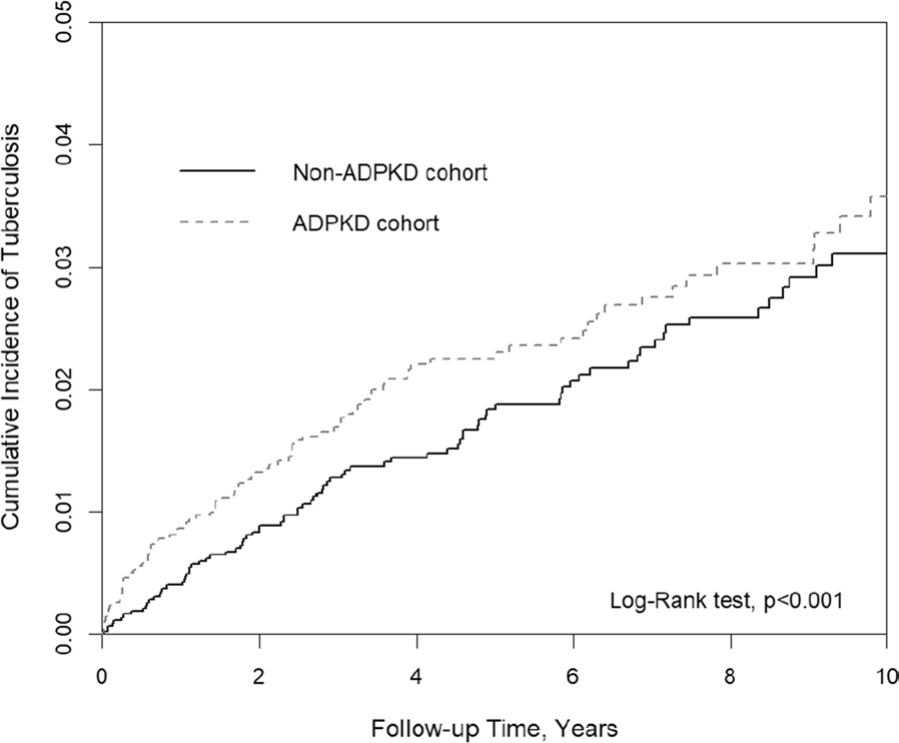
Cumulative incidence of *Mycobacterium tuberculosis* infection in the PKD and non-PKD cohorts, which reached a statistical significance (p < 0.001)

We defined two cohorts in this study, namely the PKD and non-PKD cohorts. Patients with PKD were selected with diagnoses of ICD-9-CM codes 753.12 and 753.13 and at least one hospitalization during 2000–2012. The index date was set as the date of PKD diagnosis. The non-PKD group comprised patients without any diagnosis of PKD and were propensity score-matched with PKD cases by age, sex, index year, and comorbidities. The index year of the control group was randomly assigned according to the index date distribution of PKD cases before matching (Fig. 1).

Comorbidities included in this study were alcohol-related diseases (ICD-9-CM codes 291, 303, 305.00, 305.01, 305.02, 305.03, 571.0–571.3, 790.3 and V11.3), chronic kidney disease (ICD-9-CM codes 580–589), chronic liver disease (ICD-9-CM codes 571.5, 571.6,V02.61, 070.20, 070.22, 070.30, 070.32,V02.62, 070.41, 070.44, 070.51,070.54,571.40, 571.41, 571.49, 571.8, and 571.9), chronic obstructive pulmonary disease (COPD; ICD-9-CM codes 491, 492, and 496), diabetes mellitus (ICD-9-CM code 250), bacterial and viral infections, pneumoconiosis (ICD-9-CM codes 50 and A326), hypertension (ICD-9-CM codes 401–405), and hyperlipidemia (ICD-9-CM code 272).

The primary outcome of interest was the diagnosis of TB infection (ICD-9-CM codes 01 and A02), and the exclusion criterion was a diagnosis of TB infection before the index date of diagnosis with PKD. All patients were followed up from the index date until the diagnosis of TB infection, withdrawal from the NHIRD, death, or December 31, 2013.

### Statistical analysis

Categorical and continuous variables are presented using numbers (percentages) and means (standard deviations), respectively. The difference in each grouped variable between the two cohorts was tested using the chi-square test; the mean age was assessed using Student’s *t*-test. The incidence rate was calculated as the number of patients newly diagnosed with TB divided by the following period (per 10,000 person-years). We used the Kaplan–Meier analysis to calculate the cumulative incidence of TB and the log-rank test to evaluate the difference in survival curves between the two cohorts. The risk of TB in the PKD and non-PKD cohorts was tested using the Cox proportional hazards model for estimation and comparison demonstrated by hazard ratios (HRs), adjusted HRs (aHRs; adjusted for demographic factors and comorbidities), and 95% confidence intervals (95% CIs). All statistical analyses were performed using SAS statistical software, version 9.4 (SAS Institute Inc., Cary, NC). The cumulative incidence curve was plotted using R software. Significance was set at a two-sided p value of < 0.05.

## Results

We enrolled 13,540 patients in total (Table 1) with 6770 patients in each cohort. In our cohort, 58% of the patients were men, and the mean age of the patients was 59.1 years. No significant differences were observed in sex, age, index year, and comorbidities between the groups (p ≥ 0.05).

**Table 1:** Demographic characteristics and comorbidities of patients with newly diagnosed PKD in Taiwan during 2000–2012

Variable	PKD			p-value
	Total	No	Yes	
	N = 13,540	n = 6770	n = 6770	
	n	n (%) / mean ± SD	n (%) / mean ± SD	
Gender				0.81
Female	5660	2823 (41.7)	2837 (41.9)	
Male	7880	3947 (58.3)	3933 (58.1)	
Age at baseline				0.99
20–34	1164	583 (8.6)	581 (8.6)	
35–49	3168	1580 (23.3)	1588 (23.5)	
50–64	3903	1945 (28.7)	1958 (28.9)	
> 65	5305	2662 (39.3)	2643 (39)	
Age, mean(SD)		59.1 (16.8)	59.1 (16.8)	0.99
Comorbidity				
Hypertension	4509	2266 (33.5)	2243 (33.1)	0.67
Hyperlipidemia	742	381 (5.6)	361 (5.3)	0.45
Alcohol related disease	161	87 (1.3)	74 (1.1)	0.30
Chronic kidney disease	2853	1427 (21.1)	1426 (21.1)	0.98
ESRD	1437	714 (10.5)	723 (10.7)	0.80
Chronic liver disease	885	454 (6.7)	431 (6.4)	0.42
COPD	801	396 (5.8)	405 (6)	0.74
Diabetes	1263	644 (9.5)	619 (9.1)	0.46
Bacterial infection	3327	1644 (24.3)	1683 (24.9)	0.44
Viral infection	1011	526 (7.8)	485 (7.2)	0.18
Pneumoconiosis	171	90 (1.3)	81 (1.2)	0.49

*COPD* chronic obstructive pulmonary disease

Chi-square test, *t*-test

Table 2 presents the incidence rates, hazard ratios, and 95% CIs of TB after stratification. Compared with the non-PKD group, the PKD group displayed a significantly higher risk of TB after adjusting for age, sex, and comorbidities (aHR = 1.91, 95% CI = 1.51–2.43). When the analysis was classified into pulmonary TB (PTB) and extrapulmonary TB (EPTB), the PKD group exhibited a significantly higher risk of EPTB (aHR = 2.44, 95% CI = 1.46–4.08) and also displayed a difference in the risk of PTB when compared with the non-PKD group (aHR = 1.69, 95% CI = 1.29–2.22). After adjusting for and stratifying by age, sex, and comorbidities, the female (aHR = 2.22, 95% CI = 1.36–3.62) and male (aHR = 1.84, 95% CI = 1.40–2.41) patients; those aged 20–49 years (aHR = 3.29, 95% CI = 1.14–9.47), 50–64 years (aHR = 2.68, 95% CI = 1.60–4.49), and > 65 years (aHR = 1.66, 95% CI = 1.25–2.19); and those with any of the comorbidities (aHR = 1.71, 95% CI = 1.30–2.26), hypertension (aHR = 1.89, 95% CI = 1.35–2.64), hyperlipidemia (aHR = 4.18, 95% CI = 1.46–11.94), and diabetes (aHR = 1.92, 95% CI = 1.01–3.66) in the PKD cohort had a significantly higher risk of TB than did their counterparts in the non-PKD cohort.

**Table 2:** Incidence rates, hazard ratio, and confidence intervals of TB infection in different stratifications

Variables	Non-PKD Cohort			PKD Cohort			PKD VS. Non-PKD	
	n = 6770			n = 6770			Crude HR	Adjusted HR
	Event	Person years	IR	Event	Person years	IR	(95% CI)	(95% CI)
Overall	114	41,183	27.68	175	35,325	49.54	1.74(1.37–2.20)***	1.91(1.51–2.43)***
PTB	90	41,243	21.82	121	35,514	34.07	1.52(1.16–2.00)**	1.69(1.29–2.22)**
EPTB	22	41,386	5.32	44	35,565	12.37	2.28(1.37–3.80)**	2.44(1.46–4.08)***
Gender								
Female	25	18,070	13.84	46	16,280	28.25	2.00(1.23–3.26)**	2.22(1.36–3.62)**
Male	89	23,114	38.51	129	19,045	67.73	1.70(1.30–2.23)***	1.84(1.4–2.41)***
Age at baseline								
20–49	5	14,842	3.37	14	14,217	9.85	2.88(1.04–7.99)*	3.29(1.14–9.47)*
50–64	21	12,306	17.06	48	10,711	44.81	2.61(1.56–4.36)***	2.68(1.6–4.49)***
> 65	88	14,035	62.70	113	10,397	108.68	1.66(1.25–2.19)***	1.66(1.25–2.19)***
Baseline comorbidity								
No	26	22,890	11.36	56	20,366	27.50	2.37(1.49–3.78)**	2.69(1.69–4.30)***
Yes	88	18,294	48.10	119	14,960	79.55	1.60(1.22–2.11)***	1.71(1.30–2.26)***
Hypertension	57	11,064	51.52	88	9052	97.22	1.84(1.32–2.56)***	1.89(1.35–2.64)***
Hyperlipidemia	5	1918	26.07	14	1500	93.35	3.56(1.28–9.90)*	4.18(1.46–11.94)**
Alcohol related disease	4	422	94.81	2	303	66.01	0.66(0.12–3.60)	1.52(0.18–13.01)
Chronic kidney disease	45	6931	64.93	53	5666	93.54	1.40(0.94–2.08)	1.44(0.97–2.15)
ESRD	14	3507	39.92	16	3030	52.80	1.28(0.62–2.62)	1.27(0.61–2.63)
Chronic liver disease	20	2158	92.67	18	1654	108.85	1.13(0.60–2.14)	1.25(0.65–2.40)
COPD	24	1583	151.66	22	1253	175.54	1.09(0.61–1.94)	1.19(0.66–2.15)
Diabetes	15	2916	51.44	25	2437	102.57	1.95(1.03–3.69)*	1.92(1.01–3.66)*
Bacterial infection	50	7999	62.51	47	6885	68.27	1.06(0.71–1.58)	1.12(0.75–1.68)
Viral infection	15	2484	60.39	22	1880	117.03	1.86(0.96–3.59)	1.82(0.93–3.57)
Pneumoconiosis	7	222	314.69	4	213	187.37	0.64(0.19–2.19)	0.69(0.17–2.72)

*IR* incidence rates, per 10,000 person-years, *HR* hazard ratio, *CI* confidence interval

Adjusted HR: adjusted for age, all comorbidities in Cox proportional hazards regression

^*^p < 0.05, **p < 0.01, ***p < 0.001

When stratified by the category of with or without comorbidity, the patients with PKD without any comorbidity had a high risk of TB (aHR = 2.69, 95% CI = 1.69–4.30). When followed within 1 year from the index date, the patients with PKD had a 2.82-fold increased risk of TB (aHR = 2.82, 95% CI = 1.79–4.43). A 1.85-fold increased risk of TB (aHR = 1.85, 95% CI = 1.17–2.93) was observed in the patients with PKD when followed for 1–3 years after adjusting for demographic factors and comorbidities (Table 3).

**Table 3:** Incidence and hazard ratio of TB infection stratified by the follow-up year

Variables	Non-PKD Cohort			PKD Cohort			PKD VS. Non-PKD	
	n = 6770			n = 6770			Crude HR	Adjusted HR
	Event	Person years	IR	Event	Person years	IR	(95% CI)	(95% CI)
Follow-up year								
< 1	26	6605	39.36	67	6268	106.89	2.69(1.71–4.23)***	2.82(1.79–4.43)***
1–3	30	11,340	26.46	47	10,261	45.81	1.73(1.10–2.74)*	1.85(1.17–2.93)**
3–5	24	8634	27.80	27	7374	36.62	1.31(0.76–2.27)	1.50(0.86–2.60)
> 5	34	14,604	23.28	34	11,423	29.77	1.27(0.79–2.05)	1.54(0.95–2.49)

*IR* incidence rates, per 10,000 person-years, *HR* hazard ratio, *CI* confidence interval

Adjusted HR: adjusted for sex, age, and all comorbidities in Cox proportional hazards regression

^*^p < 0.05, **p < 0.01, ***p < 0.001

## Discussion

TB infection has become a global healthy issue. In 2018, TB had affected 10 million individuals, of whom 1.5 million died, thus imposing a massive burden on the public health system according to the World Health Organization (WHO) [20]. Because of early diagnosis and direct observation of treatment by using anti-tuberculosis agents with advanced public health services in Taiwan, a decreasing trend in TB infection was observed; the case rate decreased from 73 cases per 100,000 population to 36.7 cases per 100,000 population from 2005 to 2019 [21].

We included a large number of patients with PKD and observed them for a long time in our study. Our findings showed a signficantly higher incidence rate of TB in the PKD group (495.4 per 100,000 person-years) than in the non-PKD group (276.8 per 100,000 person-years). In the multivariate analysis after adjusting for confounders, namely sex, age, index year, and comorbidities, the PKD cohort had a 1.91-fold higher risk of TB than did the non-PKD cohort. When stratified by sites involving TB, such as pulmonary (PTB) and extrapulmonary (EPTB) sites, a 3.40-fold higher risk of EPTB was observed in the patients with PKD (aHR = 2.44, 95% CI = 1.46–4.08). Similarily, a 2.69-fold higher risk of TB was observed in the patients with PKD after excluding any underlying diseases, including diabetes, alcohol consumption, and liver disease, which may contribute to TB infection in the general population. Our findings suggest that PKD is associated with a higher TB infection risk, particularly EPTB infection, which has never been known previously. In addition, our findings may provide indirect evidence implicating PKD underlying abnormal immunological function.

Risk factors for TB infection can be divided into impaired host immunity and increased exposure. For example, impaired immunity with HIV infection poses a high risk of TB infection, including EPTB or miliary TB infection, and a low CD4 lymphocyte count is a key risk factor for TB relapse [22,23]. Several predisposing factors are associated with TB infection. The risk of TB infection is high in patients with diabetes [24], solid organ transplant [25], renal disease [26], COPD [27], hematologic malignancies [28], diseases under long-term glucocoticoid treatment or tumor necrosis factor treatment [29,30], undernutrition [31], or smoking and alcohol consumption [32,33].

TB is prevalent worldwide, and male patients are particularly prone. Male patients have an approximately two-fold increased risk of TB infection [20]. Compared with female patients, more social exposure in male patients accounted for these findings. In the present study, the male patients in the PKD group were associated with an 1.8-fold higher risk of TB infection than did those in the control group, which supports the previous findings. Male sex is supposed to be associated with more rapid progression in *PKD1*-gene mutant patients with ADPKD [34]. Notably, the observation that female patients with PKD have a 2.2-fold increased risk of TB infection needs to be eluciated further.

Underlying comorbidities in PKD patients can aggravate the risk of TB infection. In this study, we observed that the PKD patients with hypertension (aHR = 1.89, 95% CI = 1.35–2.64), hyperlipidemia (aHR = 4.18, 95% CI = 1.46–11.94), and diabetes mellitus (aHR = 1.92, 95% CI = 1.01–3.66) had a high risk of TB infection; however, those with alcohol-related disease, chronic kidney disease, chronic liver disease, COPD, diabetes, bacterial infection, viral infection, and pneumoconiosis did not have a significantly increased risk of TB infection in the study.

In cases with TB infection in renal transplantations, more than one-third of the renal transplant patients developed TB infection during the first year post- transplantation [35]. High-dose immunosuppresant agents after the initial renal transplantation might interfere with T-cell function; for example, cyclosporin suppressed purified protein derivative-specific CD4 T-cell reactivity and the production of interleukin-2 and interferon-gamma [36].

Similarily, patients with PKD in the first year of follow-up have a high risk of TB, which implies that underlying immunodysregulation may likely contribute to the findings of our study.

## Strengths and limitations

Our data were robust; however, some limitations must be addressed. First, laboratory data could not be obtained from the NHIRD. Some confounders associated with the risk of TB infection, such as tobacco and alcohol consumption, could not be measured. To overcome the inherent potential bias, proxy variables, including COPD incidence for smoking, hypertension, hyperlipidemia, and diabetic incidence for obesity, were included to reduce the bias effect of these confounders.

Second, because universal Bacillus Calmette–Guerin (BCG) vaccination has been offically implemented in Taiwan, EPTB is easily missed in daily practice. Only patients with definitive TB infection were recruited and those with subclinical infections could have been excluded from the study, resulting in the possible underestimation of the results. Third, data of imaging and tissue examinations and details regarding EPTB sites could not be obtained in the study.

## Conclusions

Taken together, our findings suggest that PKD is associated with a 1.91-fold increased risk of TB infection. Medical professionls should maintain a high index of suspicion in daily practice when treating patients with PKD, particularly those with extrapulmonary TB infection.

## Data Availability

The dataset used in this study is held by the Taiwan Ministry of Health and Welfare (MOHW). The Ministry of Health and Welfare must approve our application to access this data. Any researcher interested in accessing this dataset can submit an application form to the Ministry of Health and Welfare requesting access. Please contact the staff of MOHW (Email: stcarolwu@mohw.gov.tw) for further assistance. Taiwan Ministry of Health and Welfare Address: No.488, Sec. 6, Zhongxiao E. Rd., Nangang Dist., Taipei City 115, Taiwan (R.O.C.). Phone: + 886-2-8590-6848. All relevant data are within the paper.

## Abbreviations

PKD: Polycystic kidney disease
TB: Tuberculosis
PTB: Pulmonary Tuberculosis
EPTB: Extrapulmonary Tuberculosis
ESRD: End-stage renal disease
PC: Polycystin
NHIRD: National Health Insurance Research Database
LHID: Longitudinal Health Insurance Database
aHR: Adjusted hazard ratio
CI: Confidence interval
ESRD: End-stage renal disease
ICD-9-CM: International Classification of Diseases, Ninth revision, Clinical Modification
